# Orthorhombic charge density wave on the tetragonal lattice of EuAl_4_

**DOI:** 10.1107/S2052252522003888

**Published:** 2022-04-29

**Authors:** Sitaram Ramakrishnan, Surya Rohith Kotla, Toms Rekis, Jin-Ke Bao, Claudio Eisele, Leila Noohinejad, Martin Tolkiehn, Carsten Paulmann, Birender Singh, Rahul Verma, Biplab Bag, Ruta Kulkarni, Arumugam Thamizhavel, Bahadur Singh, Srinivasan Ramakrishnan, Sander van Smaalen

**Affiliations:** a Laboratory of Crystallography, University of Bayreuth, 95447 Bayreuth, Germany; b Department of Quantum Matter, Hiroshima University, 739-8530, Higashi-Hiroshima, Japan; c Department of Physics, Materials Genome Institute and International Center for Quantum and Molecular Structures, Shanghai University, Shanghai 200444, People’s Republic of China; d P24, PETRA III, Deutsches Elektronen-Synchrotron DESY, Notkestrasse 85, 22607 Hamburg, Germany; e Mineralogisch-Petrographisches Institut, Universität Hamburg, 20146 Hamburg, Germany; f Department of Condensed Matter Physics and Materials Science, Tata Institute of Fundamental Research, Mumbai 400005, India

**Keywords:** inorganic materials, density functional theory, aperiodic structures, phase transitions, charge density wave, twinning, modulated, superspace

## Abstract

EuAl_4_ possesses the BaAl_4_ crystal structure type with tetragonal symmetry *I*4/*mmm*. It undergoes a charge density wave (CDW) transition at *T*_CDW_ = 145 K and features four consecutive antiferromagnetic phase transitions below 16 K. Here we use single-crystal X-ray diffraction to determine the incommensurately modulated crystal structure of EuAl_4_ in its CDW state. The CDW is shown to be incommensurate with modulation wave vector **q** = (0,0,0.1781 (3)) at 70 K. The symmetry of the incommensurately modulated crystal structure is orthorhombic with superspace group *Fmmm*(00σ)*s*00, where *Fmmm* is a subgroup of *I*4/*mmm* of index 2. Both the lattice and the atomic coordinates of the basic structure remain tetragonal. Symmetry breaking is entirely due to the modulation wave, where atoms Eu and Al1 have displacements exclusively along *a*, while the fourfold rotation would require equal displacement amplitudes along *a* and *b*. The calculated band structure of the basic structure and interatomic distances in the modulated crystal structure both indicate the Al atoms as the location of the CDW. The tem­per­ature dependence of the specific heat reveals an anomaly at *T*_CDW_ = 145 K of a magnitude similar to canonical CDW systems. The present discovery of orthorhombic symmetry for the CDW state of EuAl_4_ leads to the suggestion of monoclinic instead of orthorhombic symmetry for the third AFM state.

## Abstract 2

The incommensurate charge density wave of EuAl_4_ below *T*_CDW_ = 145 K is found to possess orthorhombic symmetry, despite an average crystal structure that remains tetragonal in very good approximation. This finding has ramifications for the interpretation of all physical properties of EuAl_4_, in particular, its multiple magnetic transitions.

## 1. Introduction

**Figure 1:**
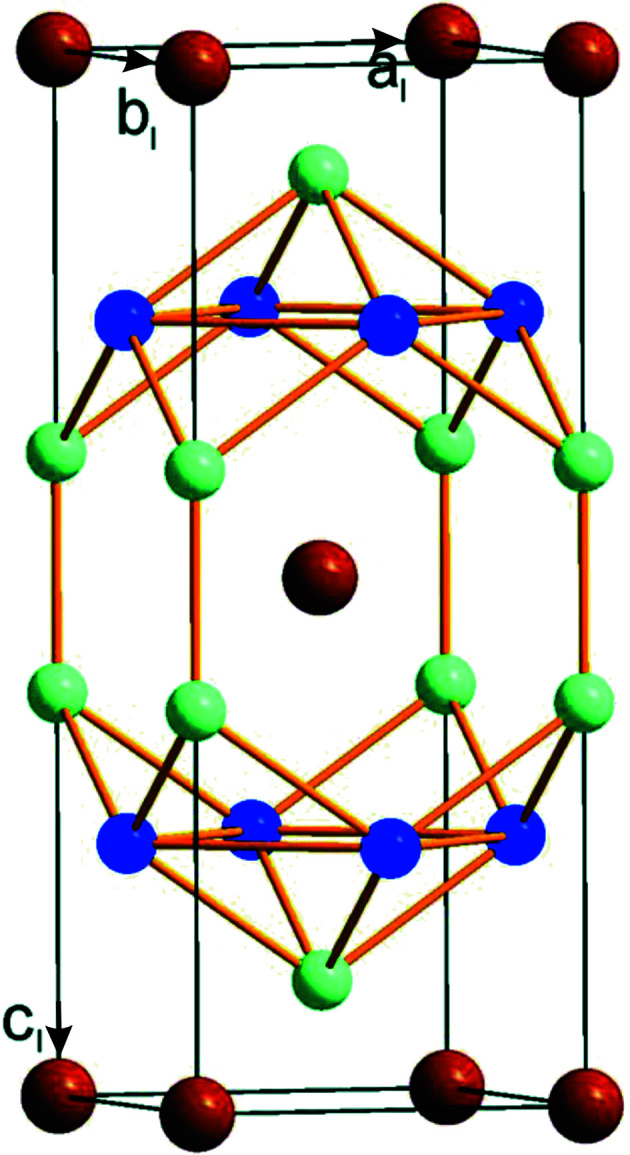
The crystal structure of EuAl_4_ with the space group *I*4/*mmm* in the periodic phase at 250 K. Depicted is the *I*-centred unit cell with basis vectors *a*_*I*_, *b*_*I*_ and *c*_*I*_. Brown spheres correspond to the Eu atoms, dark-blue spheres represent Al1 atoms and green spheres stand for Al2 atoms. The shortest interatomic distances are: *d*(Eu—Eu) = 4.3949 (2) Å, *d*(Al1—Al1) = 3.1077 (1) Å, *d*(Al2—Al1) = 2.664 (1) Å and *d*(Al2—Al2) = 2.568 (4) Å.

EuAl_4_ has attracted attention because it develops a charge density wave (CDW) below *T*_CDW_ = 145.1 K and exhibits four successive magnetic transitions below 16 K (Nakamura *et al.*, 2015 ▸; Shimomura *et al.*, 2019 ▸). EuAl_4_ adopts the BaAl_4_ structure type that has symmetry according to the tetragonal space group *I*4/*mmm* (Parthé *et al.*, 1983 ▸; Nakamura *et al.*, 2015 ▸), as shown in Fig. 1 ▸. It belongs to a large family of isostructural compounds, including magnetic EuGa_4_, fully ordered EuAl_2_Ga_2_ and nonmagnetic SrAl_4_ and BaAl_4_ (Nakamura *et al.*, 2016 ▸; Stavinoha *et al.*, 2018 ▸; Ōnuki *et al.*, 2020 ▸). Recently, a symmetry-protected nontrivial topology of the electronic band structure was proposed for BaAl_4_ (Wang *et al.*, 2021 ▸). In the case of magnetic EuAl_4_, a chiral spin structure, like skyrmions reported in some divalent Eu compounds, such as EuPtSi (Kaneko *et al.*, 2019 ▸; Ōnuki *et al.*, 2020 ▸), was proposed on the basis of the observation of the topological Hall resistivity and muon-spin rotation and relaxation (μ*SR*) studies (Shang *et al.*, 2021 ▸; Zhu *et al.*, 2022 ▸). If such a nontrivial texture was confirmed, EuAl_4_ would represent a rare case of a compound where one could observe the co-existence of exotic magnetic order and a CDW. Since these exotic electronic and spin structures depend on the symmetry, knowledge of the true symmetry of the crystal structure is thus of utmost importance for understanding non­trivial magnetic properties. Here we show that the CDW transition of EuAl_4_ is accompanied by a lowering of the symmetry towards orthorhombic, and we present the incommensurately modulated CDW crystal structure.

EuAl_4_ is one of a few compounds (Schutte *et al.*, 1993 ▸) where the lowering of the crystal symmetry at a phase transition is governed by the symmetry of the incommensurate modulation wave describing the CDW, while any lattice distortion could not be detected in the present high-resolution diffraction experiment with synchrotron radiation. This feature might explain why the lowering of symmetry has not been found in earlier studies on EuAl_4_.

This behaviour is in contrast to other CDW materials, like Er_2_Ir_3_Si_5_, Lu_2_Ir_3_Si_5_ and BaFe_2_Al_9_, for which the CDW transitions are accompanied by large lattice distortions (Ramakrishnan *et al.*, 2020 ▸, 2021 ▸; Meier *et al.*, 2021 ▸).

The phenomenon of CDW was originally identified as a property of crystals with quasi-one-dimensional (1D) electron bands, such as NbSe_3_ and K_0.3_MoO_3_ (Gruner, 1994 ▸; Monceau, 2012 ▸). A CDW is formed due to Fermi surface nesting (FSN), where the nesting vector of the periodic structure becomes the wave vector of the CDW of the metallic bands, as well as of the accompanying modulation of the atomic positions (periodic lattice distortion or PLD). The modulation wave vector can be commensurate or incommensurate with respect to the underlying periodic basic structure. More recent research has found that CDWs can develop in crystalline materials that lack the 1D property of their crystal structures and rather possess three-dimensionally (3D) structured electron bands. Alternate mechanisms have been proposed for the formation of CDWs in 3D compounds, including the mechanism of *q*-dependent electron–phonon coupling (EPC) (Zhu *et al.*, 2015 ▸, 2017 ▸). Strongly correlated electron systems may also support the formation of CDWs (Chen *et al.*, 2016 ▸). The latter are often found for rare-earth-containing intermetallic compounds, like the series of isostructural compounds *R*_5_Ir_4_Si_10_ (*R* = rare earth) (Ramakrishnan & van Smaalen, 2017 ▸).

The interplay between CDWs and magnetism in rare-earth compounds continues to attract attention. A competition between these two symmetry-breaking phenomena can be expected, because both CDWs and magnetic order depend on the Fermi surface through FSN and the Ruderman–Kittel–Kasuya–Yosida (RKKY) interaction between the localized magnetic moments of the 4*f* electrons, as it is found for EuAl_4_ (Kobata *et al.*, 2016 ▸). In the case of magnetoelastic coupling, the lattice distortion may couple to the PLD or the lattice distortion in the CDW state or to the EPC. Experimentally, the co-existence of a CDW and antiferromagnetic (AFM) order has been established for Er_5_Ir_4_Si_10_ and Sm_2_Ru_3_Ge_5_ (Galli *et al.*, 2002 ▸; Kuo *et al.*, 2020 ▸). The series of compounds *R*NiC_2_ (*R* = Pr, Nd, Gd, Tb, Dy, Ho or Er) exhibit both CDW and AFM phase transitions (Roman *et al.*, 2018 ▸; Shimomura *et al.*, 2016 ▸; Kolincio *et al.*, 2017 ▸; Maeda *et al.*, 2019 ▸). SmNiC_2_ is an exception in this series, since it develops ferromagnetic (FM) order below *T*_*C*_ = 17.7 K, at which transition the CDW is destroyed (Shimomura *et al.*, 2009 ▸; Wölfel *et al.*, 2010 ▸). Recently, co-existence of CDW and FM orders was found for the field-induced FM state of TmNiC_2_ (Kolincio *et al.*, 2020 ▸).

Magnetism of EuAl_4_ is related to localized magnetic moments of the Eu atoms in their divalent state: the electronic configuration 4*f* ^7^ implies *J* = *S* = 7/2 and *L* = 0, where *J* is the total angular momentum, *S* is the spin angular momentum and *L* is the orbital angular momentum (Wernick *et al.*, 1967 ▸; Nakamura *et al.*, 2015 ▸). This allows the study of the collective magnetism without single-ion anisotropy, as divalent Eu has zero orbital angular momentum. Magnetic interactions are governed by the RKKY interaction (Nakamura *et al.*, 2015 ▸). Neutron diffraction has established that the AFM order involves an incommensurate modulation wave (Kaneko *et al.*, 2021 ▸). Single-crystal X-ray diffraction (SXRD) has shown the co-existence of the incommensurate CDW modulation and AFM order (Shimomura *et al.*, 2019 ▸). Furthermore, Shimomura *et al.* (2019) ▸ proposed a lowering of the lattice symmetry at the third magnetic transition towards *Immm* orthorhombic. This is essentially different from the present discovery of *Fmmm* orthorhombic symmetry below the CDW transition.

EuAl_4_ possesses a 3D band structure with localized 4*f* electrons of Eu well below the Fermi surface (Kobata *et al.*, 2016 ▸). The CDW mainly involves orbitals of the Al atoms (Kaneko *et al.*, 2021 ▸). This is in agreement with the observation of a CDW with *T*_CDW_ = 243 K in isostructural SrAl_4_, where nonmagnetic Sr replaces Eu (Nakamura *et al.*, 2016 ▸; Niki *et al.*, 2020 ▸). Here we present electronic band structure calculations for the tetragonal structure of EuAl_4_. They confirm that the location of the CDW is on the Al atoms. They reveal a 3D band structure with a highly structured Fermi surface.

## 2. Experimental and computational details

Single crystals of EuAl_4_ were synthesized by the Al self-flux method. The elements europium (Lieco, 99.9% purity) and aluminium (Alfa Aesar, 99.999%) were filled into an alumina crucible in the ratio 1:20. The crucible was sealed in an evacuated quartz glass ampoule. It was heated to a tem­per­ature of 1323 K and held at this tem­per­ature for 24 h. The crucible was then cooled to 1073 K over a period of 6 h and then cooled slowly at a rate of 2 K h^−1^ to 923 K, at which point the crystals were separated from the molten metal by centrifugation. The 1:4 stoichiometry of the product was confirmed by energy-dispersive X-ray spectroscopy (EDX), as well as by structure refinement against SXRD data.

X-ray diffraction experiments were performed at Beamline P24 of PETRA III at DESY in Hamburg, employing radiation of wavelength 0.5000 Å. The tem­per­ature of the specimen was controlled by a CRYOCOOL open-flow helium gas cryostat. Complete data sets of the intensities of the Bragg reflections were measured at tem­per­atures of 250 K (tetragonal phase) and of 70 and 20 K (CDW phase). Each run of data collection comprises 3640 frames, corresponding to a rotation of the crystal over 364°, which was repeated 10 times. These data were binned to a data set of 364 frames of 1° of rotation and 10 s exposure time, using the *SNBL* toolbox (Dyadkin *et al.*, 2016 ▸); see §S1 in the supporting information.

The *EVAL15* software suite (Schreurs *et al.*, 2010 ▸) was used for processing the SXRD data. At 250 K, a single run was collected at a crystal-to-detector distance of 110 mm and without a 2θ offset of the detector. At 70 and 20 K, a crystal-to-detector distance of 260 mm required two runs, with and without 2θ offset, respectively. The two binned runs for 70 K and those for 20 K were integrated separately, and subsequently merged in the module ANY of *EVAL15*. *SADABS* (Sheldrick, 2008 ▸) was used for scaling and absorption correction with Laue symmetry 4/*mmm* for the 250 K data and *mmm* for the 70 and 20 K data. The reflection file produced was imported into *JANA2006* (Petricek *et al.*, 2014 ▸, 2016 ▸). Table 1 ▸ gives the crystallographic information.

The magnetic susceptibility χ(*T*) has been measured for tem­per­atures in the range 2.5–300 K, using a commercial SQUID magnetometer (MPMS5 by Quantum Design, USA). Measurements were made in fields of 0.1 and 0.5 T.

The specific heat, *C*_*p*_(*T*), was measured from 220 to 8 K by the thermal relaxation method, using a physical property measuring system (PPMS, Quantum Design, USA).

Density functional theory (DFT)-based calculations were performed within the generalized gradient approximation (GGA) using the projector augmented (PAW) wave method, as implemented in the Vienna *Ab-initio* Simulation Package (VASP) (Kresse & Joubert, 1999 ▸; Kresse & Furthmüller, 1996 ▸). The Perdew–Burke–Ernzerhof (PBE) functional was used to consider the exchange–correlation effects (Perdew *et al.*, 1996 ▸). An energy cut-off of 380 eV was used for the plane-wave basis set, and a Γ-centred 9 × 9 × 9 *k* mesh was employed for the bulk Brillouin zone sampling. Spin-orbit coupling effects were considered in all the calculations. We employed Eu^2+^ by considering the remaining 4*f* electrons as core electrons. A tight-binding Hamiltonian was generated to compute the Fermi surface on a finer *k* grid (Marzari & Vanderbilt, 1997 ▸). The *FermiSurfer* software package was used to visualize the Fermi surface (Kawamura, 2019 ▸).

## 3. Discussion

### 3.1. Analysis of the CDW structure

**Figure 2:**
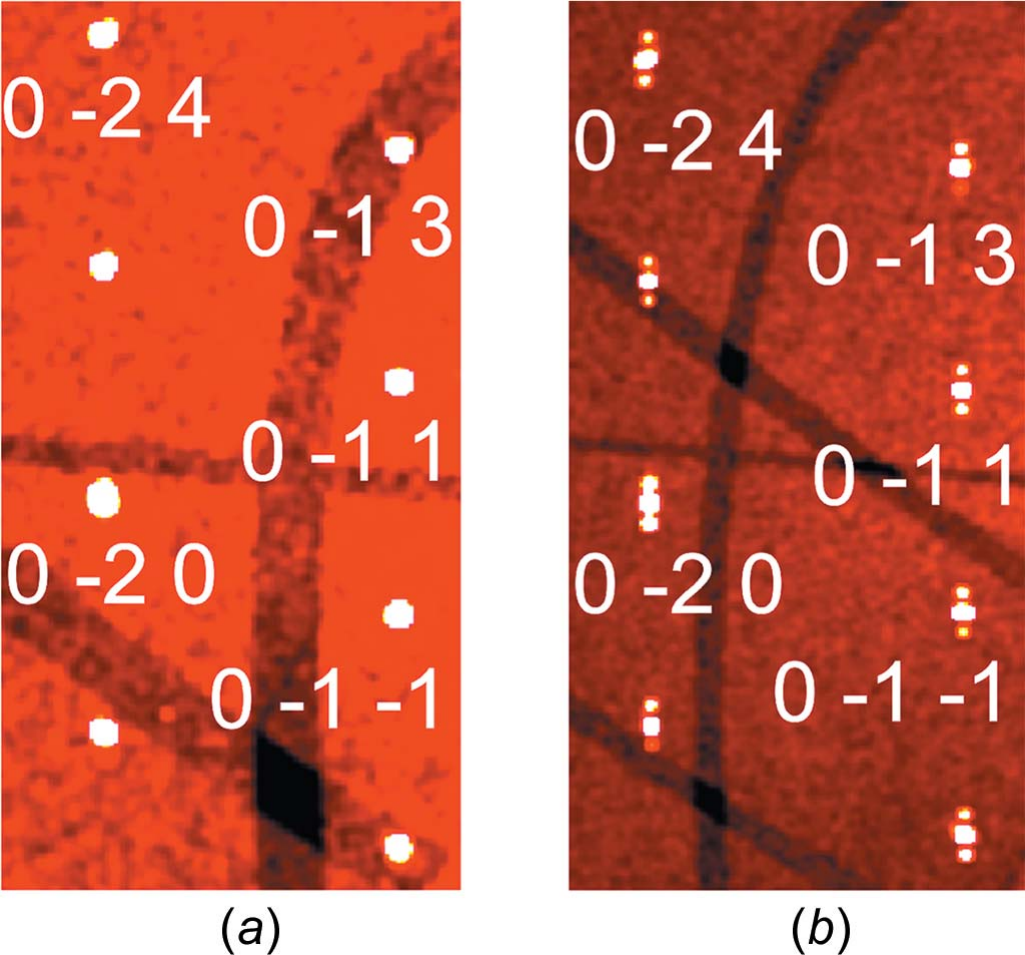
Excerpt of the reconstructed reciprocal layer (0,*k*,*l*) for SXRD data measured at (*a*) *T* = 250 K and (*b*) *T* = 70 K. Indices are given for several main reflections. Panel (*b*) is better resolved than panel (*a*) because of the longer crystal-to-detector distance at 70 K. Dark bands are due to insensitive pixels between the active modules of the PILATUS3 X CdTe 1M detector.

SXRD at 250 K confirmed the *I*4/*mmm* crystal structure of EuAl_4_. The SXRD data at 70 K revealed satellite reflections at positions that can be described by the modulation wave vector **q** = (0,0,0.1781 (3)), in agreement with the results of Nakamura *et al.* (2015) ▸ and Shimomura *et al.* (2019) ▸. Visualization of the SXRD data was done with aid of *CrysAlis PRO* (Rigaku OD, 2019 ▸). Figs. 2 ▸(*a*) and 2 ▸(*b*) show a small part of the (0,*k*,*l*) plane of the reconstructed reciprocal lattice. Satellite reflections along c* are clearly visible at 70 K [Fig. 2 ▸(*b*)]. Upon further cooling to 20 K, there is a reduction by 0.004 in the σ_3_ component of the modulation wave vector, in agreement with Shimomura *et al.* (2019) ▸. Higher-order satellite reflections have not been observed, probably because of the limited dynamic range in our experiment [compare to Shimomura *et al.* (2019) ▸].

We note that the lattice parameters in the CDW phase do not give evidence for an orthorhombic lattice distortion. This could explain why earlier work has not found this symmetry lowering.

In order to determine the crystal structure of the CDW phase, we have tested different superspace groups for its symmetry (see Table S2 in the supporting information). It is noticed that the tetragonal lattice allows two fundamentally different orthorhombic lattices as subgroups: *Immm* preserves the mirror planes perpendicular to the *a* and *b* axes of *I*4/*mmm*, while *Fmmm* preserves the diagonal mirror planes. These properties also explain the different numbers of unique reflections with correspondingly different redundancies for averaging in the two symmetries, because coverage of reciprocal space is incomplete in the present experiment. Table 2 ▸ provides the crystallographic data for three of the refined crystal structures (compare Table S2 in the supporting information). The three models, *i.e.* A, B and C, are discussed below.

#### 3.1.1. Model A

In the diffraction pattern, we did not observe any splitting of the main or satellite reflections, where split reflections would indicate a twinned crystal of lower symmetry. Also, we did not find a distortion in the lattice parameters, as it would occur for a single-domain crystal of lower symmetry. Furthermore, the preservation of tetragonal symmetry within the CDW phase was reported in the literature (Nakamura *et al.*, 2015 ▸; Shimomura *et al.*, 2019 ▸). Therefore, initial data processing was performed under the assumption of tetragonal symmetry, employing point group 4/*mmm* for scaling and absorption correction in *SADABS* (Sheldrick, 2008 ▸). Structure refinements of the incommensurately modulated structure were performed with a model with superspace group *I*4/*mmm*(00σ)0000. Table 2 ▸ shows that *R*_int_, as well as *R*_*F*_, for the main reflections are reasonably low, indicating that the average structure of the CDW phase is still tetragonal in good approximation. This conclusion is reinforced by the fact that refinement of the average structure against main reflections leads to an *R*_*F*_ value of 1.55%, an excellent fit. However, the *R*_*F*_ value is 60.46% for the satellite reflections. This high value indicates that the satellite reflections are not well fitted and that the CDW modulation does not have tetragonal symmetry.

This makes Model A an unsuitable candidate for the incommensurate CDW structure. Other tetragonal superspace groups were also tested, leading to similar failures in describing the modulation wave or with reflection conditions violated by the measured SXRD data (see Table S2 in the supporting information). As a result, we can rule out tetragonal symmetry for the modulated CDW crystal structure.

#### 3.1.2. Model B

As a second model we considered a lowering of the symmetry from tetragonal *I*4/*mmm* to its orthorhombic subgroup *Immm*. This orthorhombic point symmetry was used for scaling and absorption correction of the SXRD data in *SADABS* (Sheldrick, 2008 ▸). The CDW phase transition allows for pseudomerohedral twinning of two differently oriented domains on the tetragonal lattice, that are related by the missing fourfold rotation (Parsons, 2003 ▸). Since split reflections or a lattice distortion could not be detected in the SXRD data, all Bragg reflections have contributions from both domains. The structure refinement of a model in superspace group *Immm*(00σ)*s*00 has lead to a twin volume ratio of 0.485:0.515, thus explaining the nearly tetragonal point symmetry of the SXRD data. *R* values indicate a good fit to the SXRD data for this model (Table 2 ▸). As a result, this model is a prime candidate for describing the incommensurately modulated crystal structure of the CDW phase.

#### 3.1.3. Model C

As the last model we present model C with symmetry according to *Fmmm*, the other orthorhombic subgroup, which now preserves the diagonal mirror planes of *I*4/*mmm*. Scaling and absorption correction of the SXRD data was performed with *SADABS* according to the differently oriented point group *mmm* (Sheldrick, 2008 ▸). Again, two domains are possible that are related by the missing fourfold rotation. The structure refinement of a model in superspace group *Fmmm*(00σ)*s*00 has lead to a twin volume ratio of 0.454 (4):0.546, thus explaining the nearly tetragonal point symmetry of the SXRD data. *R* values indicate an excellent fit to the SXRD data for this model (Table 2 ▸), which is significantly better than that of model B. Furthermore, the refined parameters possess slightly smaller s.u. values in model C than in model B, while the number of parameters is one smaller in model C (Tables S4 and S5 in the supporting information). Therefore, the best fit to the SXRD data has been obtained for a modulated crystal structure with symmetry according to the superspace group *Fmmm*(00σ)*s*00. *Fmmm*(00σ)0*s*0 is an alternate setting of this superspace group, while all other symmetries lead to a worse fit to the SXRD data (Table S2 in the supporting information).

**Figure 3:**
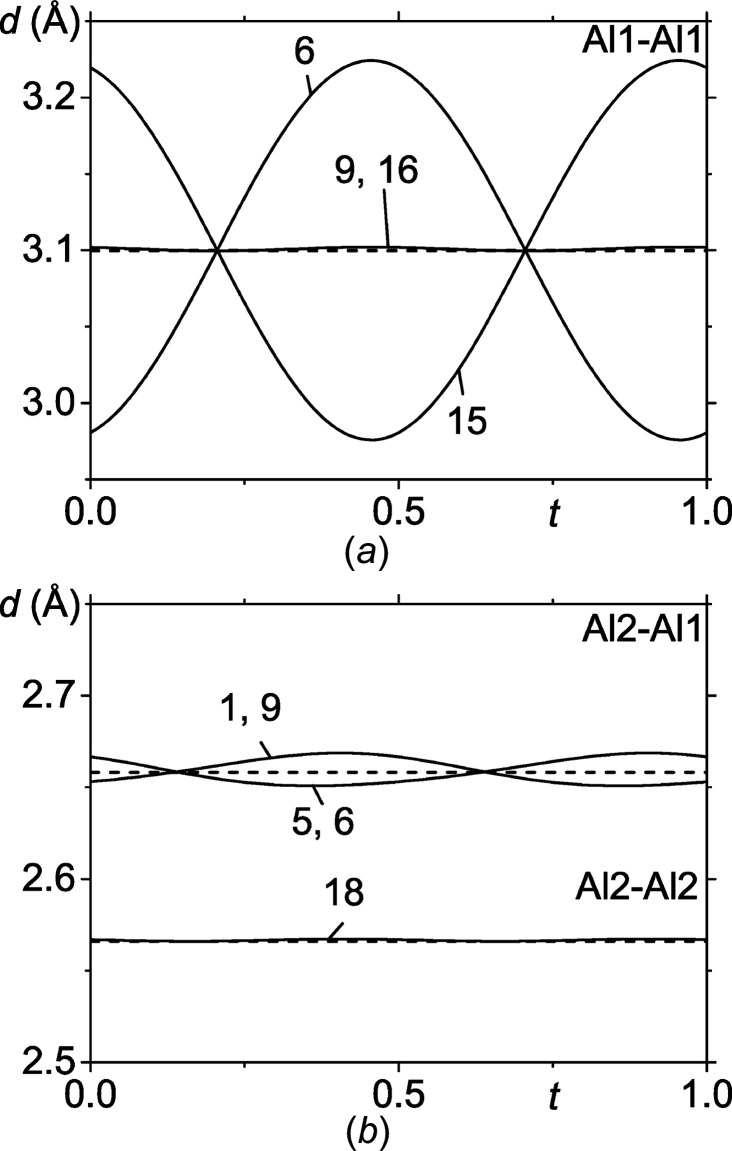
*t*-Plot of the interatomic distances (Å) *d*(Al1—Al1), *d*(Al2—Al1) and *d*(Al2—Al2) at 70 K, where the first atom is the central atom. The number on each curve is the number of the symmetry operator that is applied to the second atom of the bond pair. Symmetry operators are listed in Table S6 in the supporting information.

Recently, Kaneko *et al.* (2021) ▸ proposed that the CDW of EuAl_4_ involves displacements of the Al atoms perpendicular to *c*, while Eu would not be involved in the PLD. The present crystal structure involves atomic modulations exclusively perpendicular to *c*, as it is enforced by the superspace symmetry (Table S5). However, the modulation amplitudes are of comparable magnitude for all three atoms, *i.e.* Eu, Al1 and Al2. Nevertheless, a non-zero modulation amplitude is not evidence by itself that the involved atom must contribute electronic states to the CDW. The atomic modulation may also be caused by the elastic coupling to other atoms that are carrying the CDW. In EuAl_4_, the shortest interatomic distances are between Al2 atoms and Al1–Al2 (Fig. 3 ▸). They are hardly modulated and form a two-dimensional (2D) network of Al perpendicular to **c** (Fig. 1 ▸). The largest modulation is found for the next shorter Al—Al distance between Al1 atoms (Fig. 3 ▸). This strong modulation suggests that the CDW resides on the layers of Al atoms. Eu is elastically coupled to Al1 and Al2 (Fig. S2) and is not part of the CDW. The *t* plots of interatomic distances cannot elaborate on the precise location: the CDW resides either on the Al1 atoms or on a network of Al1 and Al2 atoms (Fig. 3 ▸).

### 3.2. Electronic structure and Fermi surface

**Figure 4:**
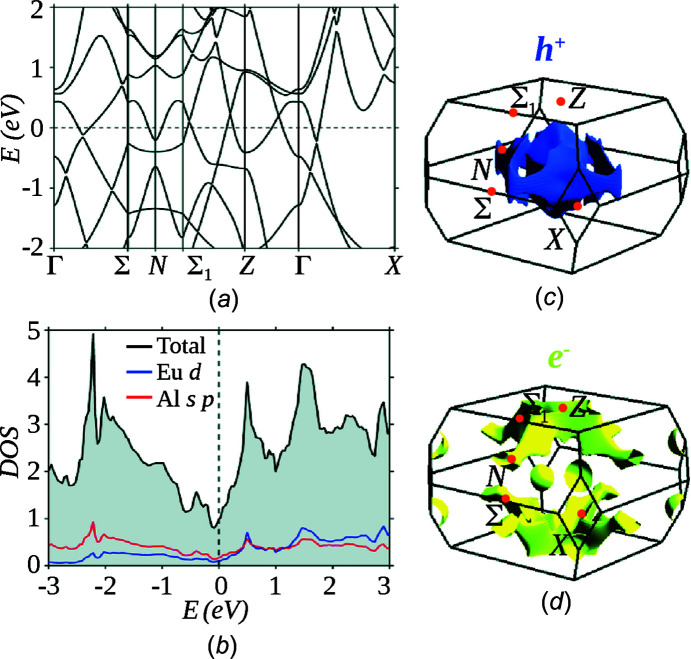
(*a*) Bulk band structure and (*b*) density-of-states (DOS) of EuAl_4_. The dashed lines in parts (*a*) and (*b*) mark the Fermi level at energy (*E*) zero. The calculated (*c*) hole (blue) and (*d*) electron (yellow) Fermi pockets in the primitive bulk Brillouin zone. Three-dimensionally (3D) structured hole and electron Fermi pockets are resolved.

Fig. 4 ▸(*a*) shows the calculated band structure along the high-symmetry directions in the primitive Brillouin zone of the periodic crystal structure of EuAl_4_ with *I*4/*mmm* symmetry. Both the valence and conduction bands cross the Fermi level *E*_*F*_, resolving its metallic ground state. Importantly, the bands along Γ–*Z* have a substantial energy dispersion. This indicates the 3D nature of the Fermi surface as discussed below. There is a Dirac nodal crossing above the Fermi level along the Γ–*Z* direction, which is protected by *C*_4*z*_ rotational symmetry. EuAl_4_ thus realizes a Dirac semimetallic state. To resolve the electronic states near *E*_*F*_, we present the atom-projected density of states (PDOS) in Fig. 4 ▸(*b*); Eu PDOS is in blue and Al PDOS is in red. The Al states are dominant at *E*_*F*_, indicating that Al atoms are predominantly metallic and more likely undergo CDW modulations, in agreement with the analysis of PLD (§3.1) and the literature. PDOS of Eu is comprised of *d* states at the Fermi level, while 4*f* states are well below *E*_*F*_, in agreement with the literature (Kobata *et al.*, 2016 ▸).

We present the calculated Fermi pockets associated with the valence (*h* ^+^) and conduction states (*e* ^−^) in Figs. 4 ▸(*c*) and 4 ▸(*d*). They reveal a hole pocket centred on Γ and an electron Fermi pocket centred on *Z*. Both Fermi pockets are highly structured. The *e* ^−^ Fermi pocket suggests the possibility of nesting perpendicular to the *c* axes. For the *h* ^+^ pocket, a possible FSN is not clearly resolved. It should however be noted that because of the 3D nature of the Fermi surface, the nesting may have a complicated structure. These results do not clearly indicate FSN as a mechanism for the formation of the CDW state in EuAl_4_.

### 3.3. Magnetic susceptibility

The tem­per­ature dependence of the magnetic susceptibility, measured with magnetic fields of 0.1 and 0.5 T, is shown for 2.4–300 K in Fig. S1 of the supporting information. Any change of the susceptibility at the CDW transition (*e.g.* as a change of Pauli paramagnetism) is masked by the large value of the paramagnetic susceptibility. However, the low-tem­per­ature data reveal an AFM transition below 16 K, which agrees with the previously published value (Nakamura *et al.*, 2015 ▸).

### 3.4. Specific heat

**Figure 5:**
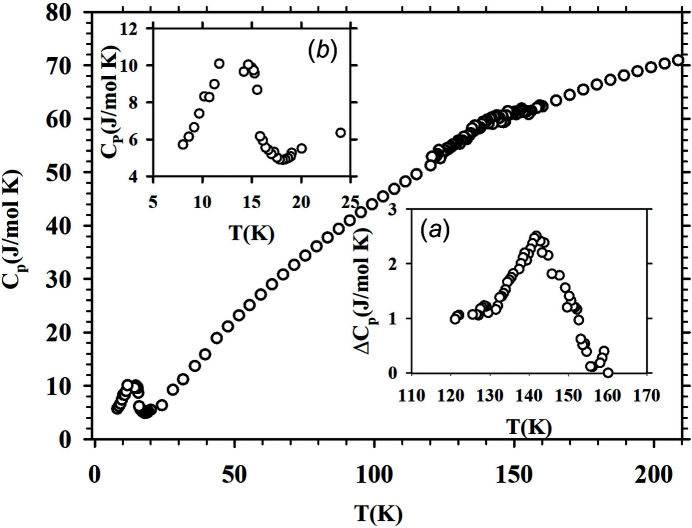
Temperature dependence of the specific heat *C*_*p*_ from 8 to 210 K. The lower inset (*a*) provides an enlarged view around the anomaly at 145 K, where Δ*C* = 2.5 J mol^−1^ K^−1^. The upper inset (*b*) displays *C*_*p*_ *versus* *T* at low tem­per­atures in the range 8–25 K.

The tem­per­ature dependence of the specific heat (*C*_*p*_) is shown for 8–210 K in Fig. 5 ▸. The high-tem­per­ature data clearly reveal a small broad jump of Δ*C*_*p*_ = 2.5 J mol^−1^ K^−1^ at 145 K, suggesting a thermodynamic phase transition (CDW) at 145 K. Such an anomaly in the tem­per­ature dependence of the specific heat is consistent with that observed in canonical CDW systems, like NbSe_3_ (Tomić *et al.*, 1981 ▸). The low-tem­per­ature data display multiple AFM transitions, which have also been observed in earlier studies (Nakamura *et al.*, 2015 ▸).

## 4. Conclusions

EuAl_4_ possesses the BaAl_4_ crystal structure type with tetragonal symmetry *I*4/*mmm*. It undergoes a CDW transition at *T*_CDW_ = 145 K. Here we have presented the incommensurately modulated crystal structure of EuAl_4_ in its CDW state. Structure refinements according to the superspace approach have shown that: (i) the modulation is incommensurate with modulation wave vector **q** = (0,0,0.1781 (3)) at 70 K, in agreement with Shimomura *et al.* (2019) ▸; and (ii) the symmetry of the CDW crystal structure is orthorhombic with superspace group *Fmmm*(00σ)*s*00, where *Fmmm* is a subgroup of *I*4/*mmm* of index 2. Despite this group–subgroup relation, we did not find any lattice distortion in the SXRD data. In addition, the atomic positions of the basic structure of *Fmmm*(00σ)*s*00 still obey the *I*4/*mmm* symmetry (Table S4). Symmetry breaking is entirely in the modulation wave (CDW and PLD), where atoms Eu and Al1 have displacements exclusively along *a* of the *F*-centred unit cell (Table S5), while the fourfold rotation would require equal displacement amplitudes along *a* and *b*.

One interesting question is the location of the CDW. Analysis of the modulation of interatomic distances (§3.1), as well as features of the electronic band structure (§3.2), have indicated the Al atoms as supporting the CDW, in agreement with the literature (Kobata *et al.*, 2016 ▸; Kaneko *et al.*, 2021 ▸).

Compounds of rare earth (*R*) and transition metals, like *R*_5_Ir_4_Si_10_ and *R*_2_Ir_3_Si_5_, contain highly correlated electron systems, with accompanying influence on the mechanism of formation of CDWs. In EuAl_4_, the majority element is the light *p*-block metal aluminium. Band structure calculations have shown that simple FSN is not a possible mechanism of CDW formation, in agreement with Kobata *et al.* (2016) ▸. Furthermore, the weak anomaly in *C*_*p*_(*T*) near *T*_CDW_ is similar to anomalies of canonical CDW materials and it is much smaller than observed for the rare-earth–transition-metal base compounds; again this would support the FSN mechanism. An alternative possibility is that the CDW is related to the nesting of nontrivial bands that are present in the band structure (Shi *et al.*, 2021 ▸; Chiu *et al.*, 2022 ▸).

The present discovery of orthorhombic symmetry for the CDW state of EuAl_4_ is important for modelling of the electronic properties of the CDW state, as well as for identifying the correct magnetic order and understanding the magnetic properties of the four AFM states below 16 K.

Recent work has used tetragonal symmetry for analysing the AFM states (Shang *et al.*, 2021 ▸; Kaneko *et al.*, 2021 ▸). Alternatively, Shimomura *et al.* (2019) ▸ have proposed orthorhombic *Immm* symmetry for the third AFM state. This orthorhombic subgroup incorporates the perpendicular mirror planes of *I*4/*mmm*, while presently found *Fmmm* is based on the diagonal mirror planes of *I*4/*mmm*. On the other hand, Shimomura *et al.* (2019) ▸ report peak splitting in neutron diffraction into ‘three maxima.’ Together with the present observation of lowering of symmetry at the CDW transition, this suggests that the third AFM state could have monoclinic symmetry (*c* unique) instead of orthorhombic symmetry, since, apparently, both the diagonal and perpendicular mirror planes are lost. Establishing the lattice symmetries in the magnetically ordered phases would require full structural analysis at tem­per­atures below 15 K.

## Supplementary Material


KGB6B0qsuYG

Crystal structure: contains datablock(s) global, I, II, III. DOI: 10.1107/S2052252522003888/gq5015sup1.cif

Structure factors: contains datablock(s) I. DOI: 10.1107/S2052252522003888/gq5015Isup2.hkl

Details on the X-ray diffraction experiment, on the choice of the symmetry of the CDW structure, and t-plots of interatomic distances. DOI: 10.1107/S2052252522003888/gq5015sup3.pdf

CIF files are provided for each of the refined crystal structures at 250 K (normal state) and at 20 and 70 K (incommensurately modulated CDW state). DOI: 10.1107/S2052252522003888/gq5015sup4.zip

CCDC references: 2165582, 2168671, 2168672

## Figures and Tables

**Table 1:** Crystallographic data of crystal A of EuAl_4_ at 250, 70 and 20 K Refinement method used: least-squares on *F*. The superspace group (SSG; No.) is given according to Stokes *et al.* (2011 ▸).

Temperature (K)	250	70	20
Crystal system	Tetragonal	Orthorhombic	Orthorhombic
Space group; SSG	*I*4/*mmm*	*Fmmm*(00σ)*s*00	*Fmmm*(00σ)*s*00
No.	139	69.1.17.2	69.1.17.2
*a* (Å)	4.3949 (1)	6.1992 (4)	6.1991 (3)
*b* (Å)	4.3949	6.2001 (4)	6.1987 (4)
*c* (Å)	11.1607 (3)	11.1477 (3)	11.1488 (4)
Volume (Å^3^)	215.57 (1)	428.47 (4)	428.41 (4)
Wavevector, *q*_*z*_	–	0.1781 (3)	0.1741 (2)
*Z*	2	4	4
Wavelength (Å)	0.50000	0.50000	0.50000
Detector distance (mm)	110	260	260
2θ-offset (°)	0	0, 25	0, 25
χ-offset (°)	−60	−60	−60
Rotation per image (°)	1	1	1
[sin(θ)/λ]_max_ (Å^−1^)	0.682610	0.748910	0.749031
Absorption, μ (mm^−1^)	5.8373	5.9090	5.9100
*T*_min_, *T*_max_	0.3211, 0.3712	0.3209, 0.3732	0.3192, 0.3676
Criterion of observability	*I* > 3σ(*I*)	*I* > 3σ(*I*)	*I* > 3σ(*I*)
Number of main reflections			
measured	1407	473	470
unique (obs/all)	109/109	174/174	176/176
Number of satellites			
measured	–	929	928
unique (obs/all)	–	279/316	263/322
*R*_int_ main (obs/all)	0.0374/0.0374	0.0136/0.0136	0.0188/0.0188
*R*_int_ sat (obs/all)	–	0.0581/0.0588	0.0606/0.0616
No. of parameters	9	18	18
*R*_*F*_ main (obs)	0.0147	0.0165	0.0213
*R*_*F*_ sat (obs)	–	0.0369	0.0311
*wR*_*F*_ main (all)	0.0214	0.0203	0.0230
*wR*_*F*_ sat (all)	–	0.0395	0.0336
*wR*_*F*_ all (all)	0.0214	0.0245	0.0250
GoF (obs/all)	1.53/1.53	1.13/1.09	0.93/0.88
Δρ_min_, Δρ_max_ (e Å^−3^)	−1.35, 1.15	−2.40, 3.58	−1.49, 1.58

**Table 2:** Crystallographic data for the three models for the modulated crystal structure at 70 K, based on different superspace groups Criterion of observability: *I* > 3σ(*I*).

Model	A	B	C
*a* (Å)	4.3834 (3)	4.3835 (3)	6.1992 (4)
*b* (Å)	4.3834	4.3841 (3)	6.2001 (4)
*c* (Å)	11.1488 (4)	11.1475 (3)	11.1477 (3)
*V* (Å^3^)	214.21 (2)	214.23 (2)	428.47 (4)
**q**	0.1782 (3)c*	0.1781 (3)c*	0.1781 (3)c*
SSG	*I*4/*mmm*(00σ)0000	*Immm*(00σ)*s*00	*Fmmm*(00σ)*s*00
*R*_int_ main (obs/all)%	1.53/1.53	1.28/1.28	1.36/1.36
*R*_int_ sat (obs/all)%	7.43/7.49	6.75/6.85	5.81/5.88
*R*_*F*_ main (obs/all)%	4.37/4.37	1.96/1.96	1.65/1.65
*R*_*F*_ sat (obs/all)%	60.46/70.53	5.25/5.83	3.69/4.05
Unique main (obs/all)	130/130	225/225	174/174
Unique sat (obs/all)	215/254	365/425	279/316
No. of parameters	13	19	18
