# Supplementary material for: Orthorhombic charge density wave on the tetragonal lattice of EuAl4
Source: IUCrJ. 2022 Apr 29;9(Pt 3):378–85. doi: 10.1107/S2052252522003888 (PMC9067112; doi:10.1107/S2052252522003888)
Supplement: Supplementary file 3 [file m-09-00378-sup3.pdf]

IUCrJ

Volume 9 (2022)

Supporting information for article:

Orthorhombic charge density wave on the tetragonal lattice of EuAl4

Sitaram Ramakrishnan, Surya Rohith Kotla, Toms Rekis, Jin-Ke Bao, Claudio Eisele, Leila Noohinejad, Martin Tolkiehn, Carsten Paulmann, Birender Singh, Rahul Verma, Biplab Bag, Ruta Kulkarni, Arumugam Thamizhavel, Bahadur Singh, Srinivasan Ramakrishnan and Sander van Smaalen

Supporting Information for

Orthorhombic charge density wave in a tetragonal lattice of $EuAl_4$

Sitaram Ramakrishnan,[a,b]* Surya Rohith Kotla,[a] Toms Rekis,[a] Jin-Ke Bao,[a,c] Claudio Eisele,[a] Leila Noohinejad,[d] Martin Tolkiehn,[d] Carsten Paulmann,[d,e] Birender Singh,[f] Rahul Verma,[f] Biplab Bag,[f] Ruta Kulkarni,[f] Arumugam Thamizhavel,[f] Bahadur Singh,[f]* Srinivasan Ramakrishnan[f]* and Sander van Smaalen [a]*

[a]*Laboratory of Crystallography, University of Bayreuth, 95447 Bayreuth Germany,* [b]*Department of Quantum Matter, Hiroshima University, 739-8530, Higashi-Hiroshima, Japan,* [c]*Department of Physics, Materials Genome Institute and International Center for Quantum and Molecular Structures, Shanghai University, Shanghai 200444, P. R. China,* [d]*P24, PETRA III, Deutsches Elektronen-Synchrotron DESY, Notkestr. 85, 22607 Hamburg, Germany,* [e]*Mineralogisch-Petrographisches Institut,Universität Hamburg, 20146 Hamburg, Germany, and* [f]*Department of Condensed Matter Physics and Materials Science,Tata Institute of Fundamental Research, Mumbai 400005, India.*

E-mail: niranj002@gmail.com, bahadur.singh@tifr.res.in, ramky07@gmail.com, smash@uni-bayreuth.de

Content

S1. X-ray diffraction at beamline P24 at DESY.

S2. Choice of symmetry.

S3. t-plots.

S1. X-ray diffraction at beamline P24 of DESY

Single-crystal X-ray diffraction (SXRD) was measured at station EH2 of beamline P24 of PETRA-III extension at DESY in Hamburg, Germany, employing radiation of a wave length of $\lambda_{P24} = 0.50000$ Å. Diffracted X rays were detected by a Pilatus 1M CdTe detector. The temperature of the sample was regulated with a CRYOCOOL open-flow cryostat, employing helium as cryo gas. Table S1 shows the thermal history of the compound along with the observed phases. The SXRD data at 250 K were measured with a crystal–to–detector distance of $D = 110$ mm. For 70 and 20 K we have collected SXRD data for two positions of the detector: $2\theta_{\rm offset} = 0$ and 25 deg, along with a long crystal–to–detector distance of 260 mm, in order to resolve the superlattice reflections. Consequently, the low-temperature data sets contain fewer (main) reflections than the data set at 250 K does.

Crystal A of dimensions $0.1 \times 0.02 \times 0.06$ mm^3 was selected for the SXRD experiment at beamline P24. Diffracted intensity was collected on the detector during rotation of the crystal by 0.1 deg and 0.1 second exposure time. Each run of data collection comprises a 10 times repeated measurement of 3640 frames, corresponding to a total rotation of the crystal by 364 deg, repeated 10 times. These data were binned to 364 frames of 1 deg of rotation and 10 seconds exposure time, using the SNBL toolbox (Dyadkin *et al.*, 2016).

Table S1. *Single-crystal X-ray diffraction of $EuAl_4$ at beamline P24 at DESY, Hamburg. For all temperatures only attenuator 12 (3640 images, 10 times repeat) is used. Entries are given in chronological order, thus providing the thermal history.*

Temperature (K)	Name	Phase
250	20f12	Periodic
70	50f12	Incommensurate
20	70f12	Incommensurate

S2. Symmetry of the modulated CDW state

Data processing of the binned data sets (Section S1) was performed with the software suite EVAL15 (Schreurs *et al.*, 2010), resulting in the lattice parameters and a list of intensities of Bragg reflections. At this point, no deviations from tetragonal symmetry could be detected.

Structure refinements have been performed with the software Jana2006 against the experimental data set (Petricek *et al.*, 2014). The modulation function for displacement modulation,

$$\mathbf{u}(\bar{x}_{s4}) = (u_x(\bar{x}_{s4}),\, u_y(\bar{x}_{s4}),\, u_z(\bar{x}_{s4}))\,, \tag{S1}$$

is defined as

$$u_\alpha(\bar{x}_{s4}) = \sum_{n=1}^{n_{max}} \{A_{n,\alpha}\sin(2\pi n\bar{x}_{s4}) + B_{n,\alpha}\cos(2\pi n\bar{x}_{s4})\}\,, \tag{S2}$$

where we have used $n_{max} = 1$ (first-order harmonics).

Structure refinements were performed with a series of structure models differing in their symmetries according to different superspace groups (Table S2). Orthorhombic symmetries were used under the assumption of twinning, thus always leading to a diffraction symmetry close to $4/mmm$.

Table S2 allows for several observations: without satellite reflections and modulation, the data are excellently fitted by the tetragonal structure model. The same basic structure is obtained for refinements in the orthorhombic superspace groups (Table S4). The only deviation from tetragonal symmetry is in the values of the ADPs.

However, satellite reflections are not well described (R_F^{obs}(sat) = 60%) or possess structure factors that are exactly zero (R_F^{obs}(sat) = 100%) for tetragonal symmetry. This can be understood from the fact that for tetragonal superspace groups the same symmetry operator, $(m, 0)$ or (m, s)—where "s" represents a phase shift of the modulation wave by half a wavelength (van Smaalen, 2012)—, exists perpendicular to **a** as well as perpendicular to **b**, or it exists perpendicular to both $\mathbf{a} \pm \mathbf{b}$. The poor fit to $Immm(00\sigma)000$ and the good fit to $Immm(00\sigma)s00$ show that the different symmetry operators are required perpendicular to **a** than perpendicular to **b**. A similar observation can be made for $Fmmm(00\sigma)s00$ and the symmetry operators (m, s) and $(m, 0)$ perpendicular to $\mathbf{a} \pm \mathbf{b}$. Obviously, tetragonal groups always include the wrong phase relation between modulation waves on different atoms. While the basic structure remains tetragonal in good approximation for all symmetries (Table S4), the modulation amplitudes for the two orthorhombic models in Table S5 illustrate the essentially different symmetry restrictions for these models.

All parameters (lower R values for a smaller number of parameters) then point to the orthorhombic superspace group $Fmmm(00\sigma)s00$ as the correct symmetry of $EuAl_4$ in its CDW state (Tables S2 and S3).

Table S2. *R values of refinements of different models (different symmetries) against SXRD at 70 K. Given are the superspace group symbol (SSG), the superspace group number (No.) in Stokes* et al. *(2011), the partial R_F value for observed main reflections, the partial R_F value for observed satellite reflections, the weighted R value for all reflections, the number of unique main reflections [N_{main} (obs/all)], the number of unique satellite reflections [N_{sat} (obs/all)] and the number of refined parameters (n_{par}). The criterion of observability is $I > 3\sigma(I)$.*

SSG	No.	R_F^{obs}(main) (%)	R_F^{obs}(sat) (%)	wR_F^{all}(all) (%)	N_{main} (obs/all)	N_{sat} (obs/all)	n_{par}
$I4/mmm(00\sigma)0000$	139.1.21.1	4.37	60.46	28.17	130/130	215/254	13
$I4/mmm(00\sigma)s0s0$	139.1.21.2	1.58	100	37.30	130/130	153/166	9
$I4/mmm(00\sigma)00ss$	139.1.21.3	1.58	100	33.47	130/130	117/119	9
$I4/mmm(00\sigma)s00s$	139.1.21.4	7.32	73.94	31.59	130/130	179/191	10
$Immm(00\sigma)000$	71.1.12.1	9.72	48.18	25.86	225/225	365/441	19
$Immm(00\sigma)s00$	71.1.12.2	1.96	5.24	3.69	225/225	365/425	19
$Immm(00\sigma)0s0$	71.1.12.2	1.96	5.24	3.69	225/225	365/425	19
$Immm(00\sigma)ss0$	71.1.12.3	4.72	100	35.37	225/225	254/284	14
$Fmmm(00\sigma)000$	69.1.17.1	4.63	59.40	27.24	174/174	279/332	17
$Fmmm(00\sigma)s00$	69.1.17.2	1.65	3.69	2.03	174/174	279/316	18
$Fmmm(00\sigma)0s0$	69.1.17.2	1.65	3.69	2.03	174/174	279/316	18
$Fmmm(00\sigma)ss0$	69.1.17.3	7.02	70.31	30.08	174/174	230/250	14

Table S3. *Crystallographic data for three models for the modulated crystal structure at 20 K, based on different superspace groups. Criterion of observability: $I > 3\sigma(I)$*

Model	A	B	C
a (Å)	4.3831(3)	4.3833(2)	6.1991(3)
b (Å)	4.3831	4.3832(2)	6.1987(4)
c (Å)	11.1484(4)	11.1483(4)	11.1488(4)
V (Å^3)	214.18(2)	214.19(2)	428.41(4)
$\mathbf{q}$	0.1742(3)$\mathbf{c}^*$	0.1741(2)$\mathbf{c}^*$	0.1741(2)$\mathbf{c}^*$
SSG	$I4/mmm(00\sigma)0000$	$Immm(00\sigma)s00$	$Fmmm(00\sigma)s00$
R_{int} main (obs/all)%	2.34/2.34	2.23/2.23	1.88/1.88
R_{int} sat (obs/all)%	7.31/7.37	7.08/7.20	6.06/6.16
R_F main (obs/all)%	5.28/5.28	2.06/2.06	2.13/2.13
R_F sat (obs/all)%	59.80/70.88	4.22/4.58	3.11/3.46
wR_F all (obs/all)%	27.84/28.43	2.69/2.73	2.48/2.50
Unique main (obs/all)	132/132	226/226	176/176
Unique sat (obs/all)	208/258	337/428	263/322
No. of parameters	13	19	18

Table S4. *Atomic coordinates x, y, z and ADPs of the basic structure of crystal A at 70 K for the models $Immm(00\sigma)s00$ and $Fmmm(00\sigma)s00$.*

Atom	x	y	z	U_{11}	U_{22}	U_{33}	U_{12}	U_{13}	U_{23}	U_{iso}^{eq}
Crystal A at $T = 70$ K, SSG: $Immm(00\sigma)s00$										
Eu1	0	0	0	0.0039(5)	0.0075(5)	0.0057(2)	0	0	0	0.0057(2)
Al1	0	0.5	0.2506(7)	0.0046(2)	0.0077(2)	0.0056(7)	0	0	0	0.0060(10)
Al2	0	0	0.3848(2)	0.0039(19)	0.0078(21)	0.0042(7)	0	0	0	0.0053(10)
Crystal A at $T = 70$ K, SSG: $Fmmm(00\sigma)s00$										
Eu1	0	0	0	0.0011(3)	0.0156(5)	0.0081(2)	0	0	0	0.0083(2)
Al1	0.25	0.25	0.25	0.0080(16)	0.0080(20)	0.0079(5)	0	0	0	0.0080(9)
Al2	0	0	0.3849(1)	0.0034(14)	0.0127(19)	0.0070(6)	0	0	0	0.0077(8)

Table S5. *Amplitudes of the modulation functions of crystal A at 70 K for $Immm(00\sigma)s00$ and $Fmmm(00\sigma)s00$. Refined values have been multiplied by the corresponding lattice parameter, in order to obtain values in Å.*

Atom	$A_{1,x}\,a$ (Å)	$A_{1,y}\,b$ (Å)	$A_{1,z}\,c$ (Å)	$B_{1,x}\,a$ (Å)	$B_{1,y}\,b$ (Å)	$B_{1,z}\,c$ (Å)
Crystal A at $T = 70$ K, SSG: $Immm(00\sigma)s00$						
Eu1	0.1282(8)	0	0	0	0	0
Al1	0.1230(31)	0	0	-0.0571(35)	0	0
Al2	0.1292(31)	0	0	0.0562(25)	0	0
Crystal A at $T = 70$ K, SSG: $Fmmm(00\sigma)s00$						
Eu1	-0.1226(5)	0	0	0	0	0
Al1	-0.1236(22)	0	0	0	-0.0621(23)	0
Al2	-0.1276(20)	0	0	-0.0575(18)	0	0

S3. Magnetic susceptibility of $EuAl_4$

The temperature dependence of the magnetic susceptibility is shown in Fig. S1 [Compare to Nakamura *et al.* (2015)].

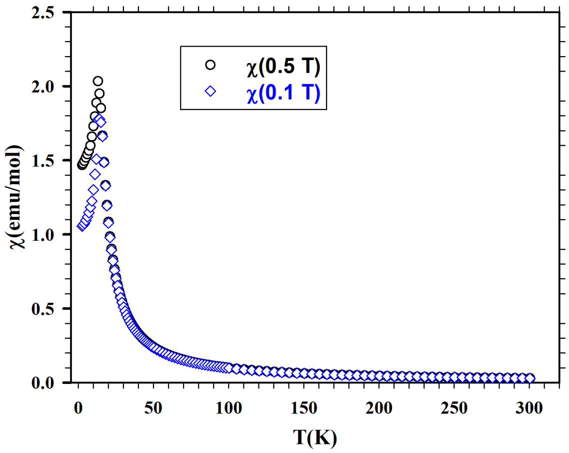

Fig. S1. Temperature dependent magnetic susceptibility of $EuAl_4$ from 2.4 to 300 K. Data measured in fields of 0.1 T and 0.5 T.

S4. *t*-plots

t-Plots of interatomic distances and of the atomic modulation functions are given for the incommesnurately modulated structure of $EuAl_4$ at 70 K in its CDW state (Figs. S1-S5). Shortest interatomic distances are between Al2 atoms (2.57 Å) and between Al1 and Al2 atoms (2.62 Å). They are hardly modulated. The strongest modulation is found for the next-nearest-neighbour Al1–Al1 contact (Fig. S2), thus suggesting that the the CDW is driven by the aluminum contributions to the band structure.

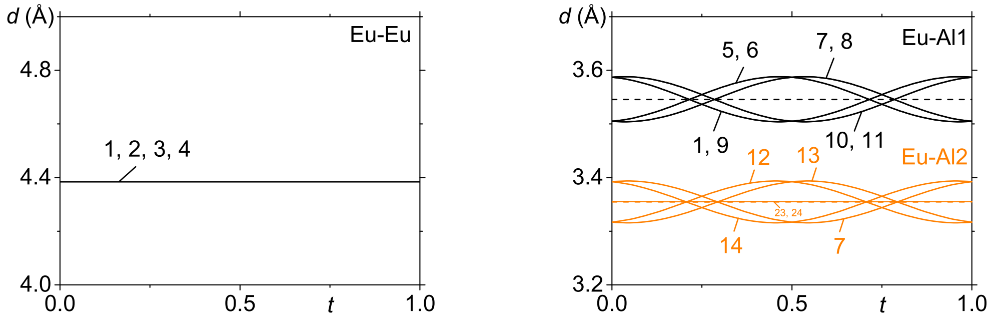

Fig. S2. *t*-Plot of interatomic distances (Å) *d*[Eu1–Eu1], *d*[Eu1–Al1] and *d*[Eu–Al2] at 70 K, where Eu is the central atom. The number on each curve is the number of the symmetry operator that is applied to the second atom of the bond pair, as listed in Table S6.

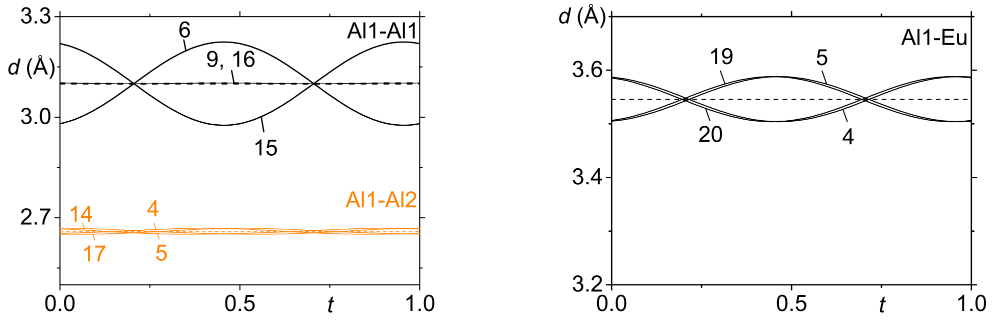

Fig. S3. t-Plot of interatomic distances (Å) d[Al1–Al1], d[Al1–Al2] and d[Al1–Eu] at 70 K, where Al1 is the central atom. The number on each curve is the number of the symmetry operator that is applied to the second atom of the bond pair, as listed in Table S6.

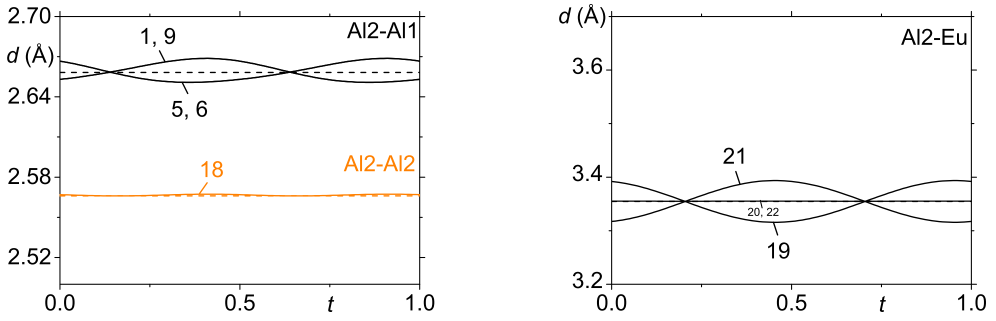

Fig. S4. t-Plot of interatomic distances (Å) d[Al2–Al1], d[Al2–Al2] and d[Al2–Eu] at 70 K, where Al2 is the central atom. The number on each curve is the number of the symmetry operator that is applied to the second atom of the bond pair, as listed in Table S6.

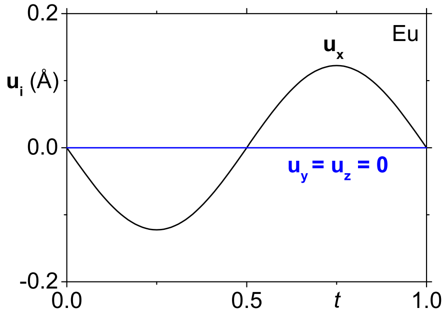

Fig. S5. t-Plot of the modulation functions (Eq. S1) for displacement modulation of the Eu atom at 70 K.

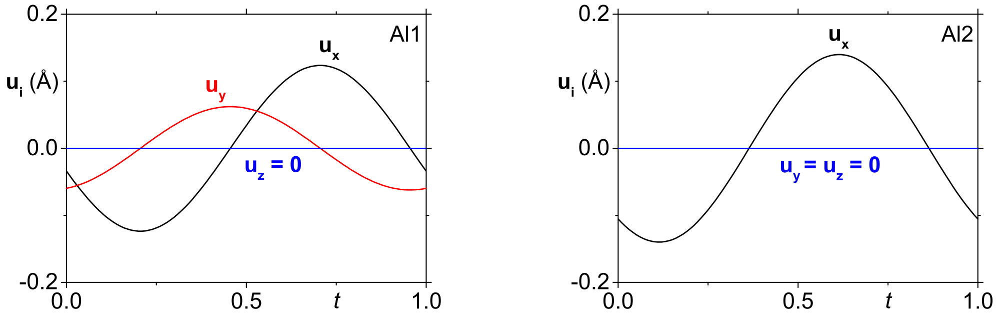

Fig. S6. t-Plot of the modulation functions (Eq. S1) for displacement modulation of Al1 and Al2 atoms at 70 K.

Table S6. *Symmetry operators for the curves of interatomic distance t-plots*

Number	Symmetry operator
1	x-1/2, y-1/2, z
2	x-1/2, y+1/2, z
3	x+1/2, y-1/2, z
4	x+1/2, y+1/2, z
5	x, y, z
6	$-x$+1/2, $-y$, $-z$+1/2
7	x-1/2, y, z-1/2
8	$-x$, $-y$, $-z$
9	$-x$, $-y$+1/2, $-z$+1/2
10	x, y-1/2, z-1/2
11	$-x$+1/2, $-y$+1/2, $-z$
12	$-x$-1/2, y, $-z$+1/2
13	x+1/2, y, z-1/2
14	$-x$+1/2, y, $-z$+1/2
15	$-x$+1/2, $-y$+1, $-z$+1/2
16	$-x$+1, $-y$+1/2, $-z$+1/2
17	$-x$,y+1/2, $-z$+1/2
18	$-x$, y, $-z$+1
19	x+1/2, y, z+1/2
20	x, y+1/2, z+1/2
21	x-1/2, y, z+1/2
22	x, y-1/2, z+1/2
23	$-x$, y-1/2, $-z$+1/2
24	$-x$, y+1/2, $-z$+1/2

References

Dyadkin, V., Pattison, P., Dmitriev, V. & Chernyshov, D. (2016). *J. Synchrotron Rad.* **23**(3), 825–829.

Nakamura, A., Uejo, T., Honda, F., Takeuchi, T., Harima, H., Yamamoto, E., Haga, Y., Matsubayashi, K., Uwatoko, Y., Hedo, M., Nakama, T. & Onuki, Y. (2015). *J. Phys. Soc. Jpn.* **84**(12), 124711.

Petricek, V., Dusek, M. & Palatinus, L. (2014). *Z. Kristallogr.* **229**, 345–352.

Schreurs, A. M. M., Xian, X. & Kroon-Batenburg, L. M. J. (2010). *J. Appl. Crystallogr.* **43**, 70–82.

van Smaalen, S. (2012). *Incommensurate Crystallography.* Oxford: Oxford University Press.

Stokes, H. T., Campbell, B. J. & van Smaalen, S. (2011). *Acta Crystallogr. A*, **67**, 45–55.
