# Supplementary material for: Orthorhombic charge density wave on the tetragonal lattice of EuAl4
Source: IUCrJ. 2022 Apr 29;9(Pt 3):378–85. doi: 10.1107/S2052252522003888 (PMC9067112; doi:10.1107/S2052252522003888)

checkCIF/PLATON report

Structure factors have been supplied for datablock(s) I

THIS REPORT IS FOR GUIDANCE ONLY. IF USED AS PART OF A REVIEW PROCEDURE FOR PUBLICATION, IT SHOULD NOT REPLACE THE EXPERTISE OF AN EXPERIENCED CRYSTALLOGRAPHIC REFEREE.

No syntax errors found. CIF dictionary Interpreting this report

Datablock: I

```
Bond precision:   Al-Al = 0.0016 A         Wavelength=1/2

Cell:               a=4.3949(1)      b=4.3949(1)      c=11.1607(3)
                    alpha=90         beta=90          gamma=90
Temperature:        250 K

                 Calculated                   Reported
Volume           215.571(11)                  215.571(9)
Space group      I 4/m m m                    I 4/m m m
Hall group       -I 4 2                       -I 4;-2
Moiety formula   Al4, Eu                      ?
Sum formula      Al4 Eu                       Al4 Eu1
Mr               259.89                       259.90
Dx,g cm-3        4.004                        4.004
Z                2                            2
Mu (mm-1)        5.853                        5.873
F000             230.0                        230.0
F000'            229.19
h,k,lmax         6,6,15                       6,6,14
Nref             111                          109
Tmin,Tmax                                     0.321,0.371
Tmin'

Correction method= # Reported T Limits: Tmin=0.321 Tmax=0.371
AbsCorr = EMPIRICAL

Data completeness= 0.982           Theta(max)= 19.960

                                                     wR2(reflections)=
R(reflections)= 0.0147( 109)                         wR= 0.0214( 109)

S = 1.530                    Npar= 9
```

```
The following ALERTS were generated. Each ALERT has the format
       test-name_ALERT_alert-type_alert-level.
Click on the hyperlinks for more details of the test.
```

Alert level B

```
PLAT031_ALERT_4_B Refined Extinction Parameter Within Range ......      1.143 Sigma
```

Alert level C

```
PLAT127_ALERT_1_C Implicit Hall Symbol  Inconsistent with Explicit    -I 4;-2
PLAT974_ALERT_2_C Check Calcd Negative Resid. Density on       Eu1      -1.26 eA-3
```

Alert level G

```
ABSMU01_ALERT_1_G  Calculation of _exptl_absorpt_correction_mu
                not performed for this radiation type.
PLAT004_ALERT_5_G Polymeric Structure Found with Maximum Dimension          2 Info
PLAT005_ALERT_5_G No Embedded Refinement Details Found  in the CIF     Please Do !
PLAT092_ALERT_4_G Check: Wavelength Given is not Cu,Ga,Mo,Ag,In Ka    0.50000 Ang.
PLAT152_ALERT_1_G The Supplied and Calc. Volume s.u. Differ by ...          2 Units
PLAT802_ALERT_4_G CIF Input Record(s) with more than 80 Characters          1 Info
PLAT808_ALERT_5_G No Parseable SHELXL Style Weighting Scheme Found     Please Check
PLAT883_ALERT_1_G No Info/Value for _atom_sites_solution_primary .     Please Do !
PLAT912_ALERT_4_G Missing # of FCF Reflections Above STh/L=  0.600          1 Note
PLAT929_ALERT_5_G No Weight Pars,Obs and Calc R1,wR2,S not Checked          ! Info
PLAT961_ALERT_5_G Dataset Contains no Negative Intensities .......     Please Check
PLAT966_ALERT_5_G Note: Non-Standard (i.e. 2.0) OMIT Threshold  of        3.0 Sig(I)
PLAT984_ALERT_1_G The Eu-f'=   -0.5035 Deviates from the B&C-Value    -0.5011 Check
PLAT985_ALERT_1_G The Eu-f"=    2.0178 Deviates from the B&C-Value     2.0030 Check
```

```
   0 ALERT level A = Most likely a serious problem - resolve or explain
   1 ALERT level B = A potentially serious problem, consider carefully
   2 ALERT level C = Check. Ensure it is not caused by an omission or oversight
  14 ALERT level G = General information/check it is not something unexpected

   6 ALERT type 1 CIF construction/syntax error, inconsistent or missing data
   1 ALERT type 2 Indicator that the structure model may be wrong or deficient
   0 ALERT type 3 Indicator that the structure quality may be low
   4 ALERT type 4 Improvement, methodology, query or suggestion
   6 ALERT type 5 Informative message, check
```

Validation response form

Please find below a validation response form (VRF) that can be filled in and pasted into your CIF.

```
# start Validation Reply Form
_vrf_PLAT031_I
;
PROBLEM: Refined Extinction Parameter Within Range ......      1.143 Sigma
RESPONSE: ...
;
# end Validation Reply Form
```

Publication of your CIF

You should attempt to resolve as many as possible of the alerts in all categories. Often the minor alerts point to easily fixed oversights, errors and omissions in your CIF or refinement strategy, so attention to these fine details can be worthwhile. In order to resolve some of the more serious problems it may be necessary to carry out additional measurements or structure refinements. However, the nature of your study may justify the reported deviations from journal submission requirements and the more serious of these should be commented upon in the discussion or experimental section of a paper or in the "special_details" fields of the CIF. *checkCIF* was carefully designed to identify outliers and unusual parameters, but every test has its limitations and alerts that are not important in a particular case may appear. Conversely, the absence of alerts does not guarantee there are no aspects of the results needing attention. It is up to the individual to critically assess their own results and, if necessary, seek expert advice.

If you wish to submit your CIF for publication in Acta Crystallographica Section C or E, you should upload your CIF via the web. If you wish to submit your CIF for publication in IUCrData you should upload your CIF via the web. If your CIF is to form part of a submission to another IUCr journal, you will be asked, either during electronic submission or by the Co-editor handling your paper, to upload your CIF via our web site.

PLATON version of 13/07/2021; check.def file version of 13/07/2021

Datablock I - ellipsoid plot

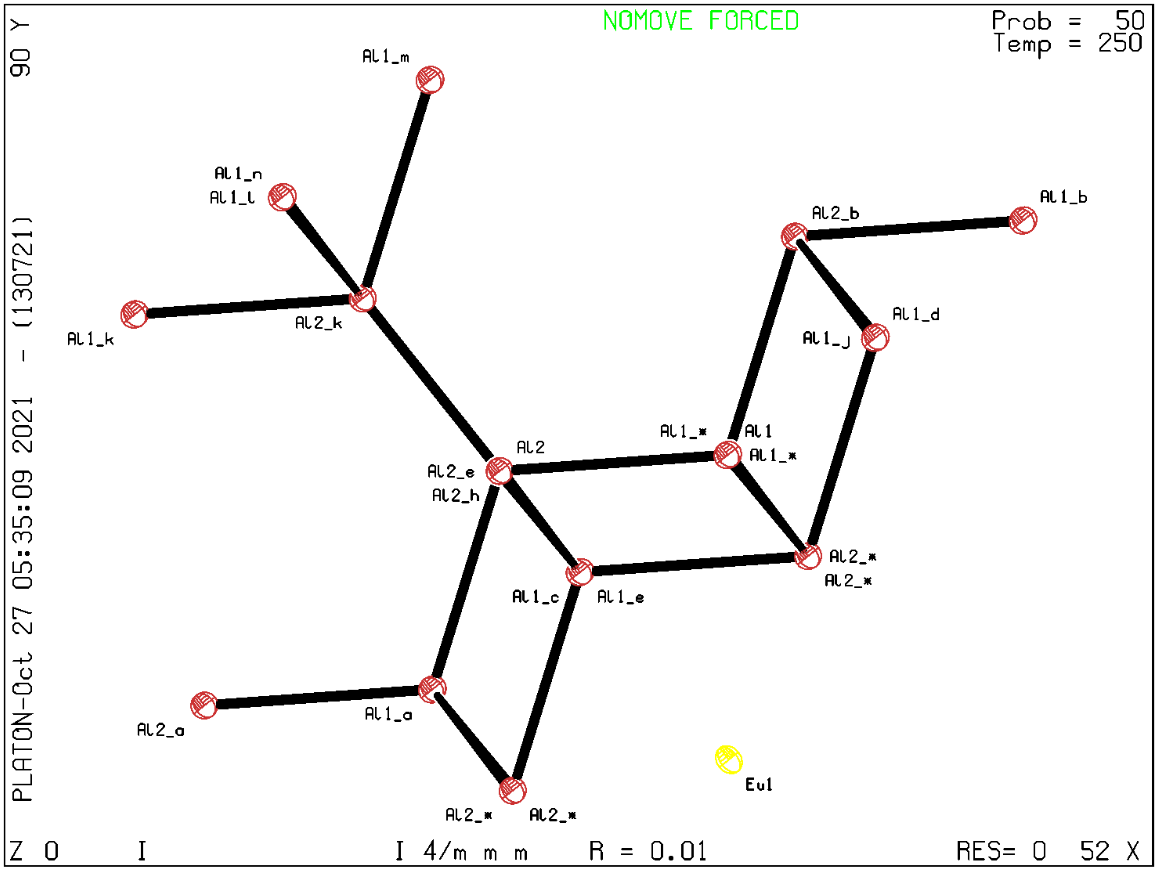

Supplement: Supplementary file 4 [file m-09-00378-sup4.zip › cif_eual4_final/250k_checkcif.pdf]
