# Supplementary material for: Orthorhombic charge density wave on the tetragonal lattice of EuAl4
Source: IUCrJ. 2022 Apr 29;9(Pt 3):378–85. doi: 10.1107/S2052252522003888 (PMC9067112; doi:10.1107/S2052252522003888)

checkCIF/PLATON report

Structure factors have been supplied for datablock(s) I

THIS REPORT IS FOR GUIDANCE ONLY. IF USED AS PART OF A REVIEW PROCEDURE FOR PUBLICATION, IT SHOULD NOT REPLACE THE EXPERTISE OF AN EXPERIENCED CRYSTALLOGRAPHIC REFEREE.

No syntax errors found. CIF dictionary Interpreting this report

Datablock: I

Bond precision: = 0.0000 A Wavelength=1/2

Cell:	a=6.1992(4)	b=6.2001(4)	c=11.1477(3)
	alpha=90	beta=90	gamma=90
Temperature:	70 K		

	Calculated	Reported
Volume	428.47(4)	428.47(4)
Space group	?	?
Hall group		?
Moiety formula	Al4, Eu2	?
Sum formula	Al4 Eu2	Al4 Eu1
Mr	411.86	259.90
Dx,g cm-3	1.596	4.029
Z	1	4
Mu (mm-1)	2.867	5.909
F000	178.0	920.0
F000’	177.09	
h,k,lmax	9,9,16	8,9,16
Nref	6020[3010]	490
Tmin,Tmax		0.321,0.373
Tmin’		

Correction method= # Reported T Limits: Tmin=0.321 Tmax=0.373
AbsCorr = EMPIRICAL

Data completeness= 0.16/0.08 Theta(max)= 21.990

R(reflections)= 0.0220(453) wR2(reflections)= wR= 0.0245(490)

S = 1.090 Npar= 18

The following ALERTS were generated. Each ALERT has the format
test-name_ALERT_alert-type_alert-level.
Click on the hyperlinks for more details of the test.

Alert level A

SYMM003_ALERT_1_A _symmetry_space_group_name_H-M is missing
Space-group symbol, including unique axis.
The following tests will not be performed.
CELLZ_01,CHEMW_03,REFLT_03,SYMMG_01,SYMMG_02

Author Response: This test is not properly performed, because CheckCif cannot handle superspace models for incommensurate structures.

SYMM004_ALERT_1_A _symmetry_equiv_pos_as_xyz loop is missing.
The symmetry equivalent positions in xyz.
The following tests will not be performed.
CELLZ_01,CHEMW_03,REFLT_03,SYMMG_01,SYMMG_02

Author Response: This test is not properly performed, because CheckCif cannot handle superspace models for incommensurate structures.

GEOM003_ALERT_1_A _geom_bond_distance is missing
Distance between atom sites 1 and 2.

Author Response: This alert appears only for 70 K andd 20 K and not 250 K data, because CheckCif cannot handle superspace models for incommensurate structures.

Alert level G

ABSMU01_ALERT_1_G Calculation of _exptl_absorpt_correction_mu
not performed for this radiation type.
PLAT814_ALERT_5_G No Validation of (In)commensurate Structure CIFs Note

3 **ALERT level A** = Most likely a serious problem - resolve or explain
0 **ALERT level B** = A potentially serious problem, consider carefully
0 **ALERT level C** = Check. Ensure it is not caused by an omission or oversight
2 **ALERT level G** = General information/check it is not something unexpected

4 ALERT type 1 CIF construction/syntax error, inconsistent or missing data
0 ALERT type 2 Indicator that the structure model may be wrong or deficient
0 ALERT type 3 Indicator that the structure quality may be low
0 ALERT type 4 Improvement, methodology, query or suggestion
1 ALERT type 5 Informative message, check

Publication of your CIF

You should attempt to resolve as many as possible of the alerts in all categories. Often the minor alerts point to easily fixed oversights, errors and omissions in your CIF or refinement strategy, so attention to these fine details can be worthwhile. In order to resolve some of the more serious problems it may be necessary to carry out additional measurements or structure refinements. However, the nature of your study may justify the reported deviations from journal submission requirements and the more serious of these should be commented upon in the discussion or experimental section of a paper or in the "special_details" fields of the CIF. *checkCIF* was carefully designed to identify outliers and unusual parameters, but every test has its limitations and alerts that are not important in a particular case may appear. Conversely, the absence of alerts does not guarantee there are no aspects of the results needing attention. It is up to the individual to critically assess their own results and, if necessary, seek expert advice.

If you wish to submit your CIF for publication in Acta Crystallographica Section C or E, you should upload your CIF via the web. If you wish to submit your CIF for publication in IUCrData, you should upload your CIF via the web. If your CIF is to form part of a submission to another IUCr journal, you will be asked, either during electronic submission or by the Co-editor handling your paper, to upload your CIF via our web site.

PLATON version of 13/07/2021; check.def file version of 13/07/2021

Datablock I - ellipsoid plot

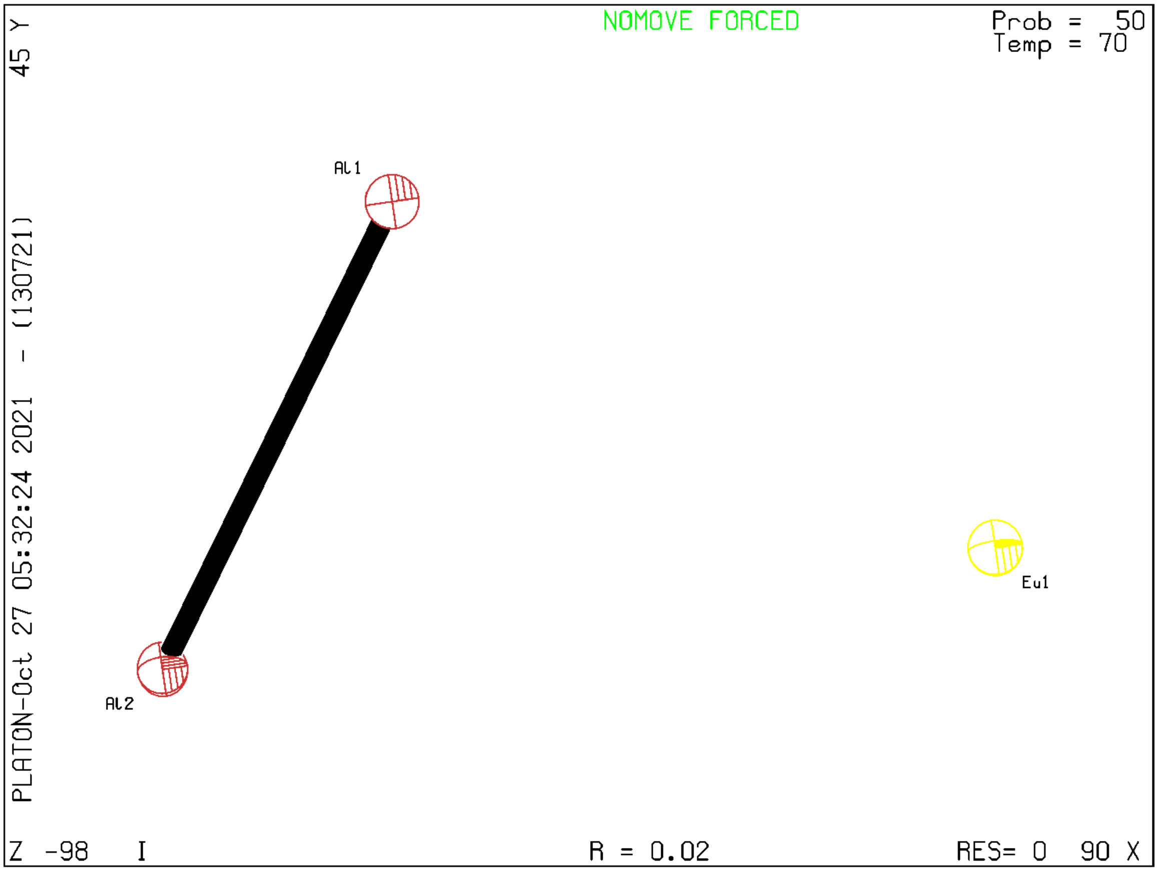

Supplement: Supplementary file 4 [file 70k_checkcif.pdf › cif_eual4_final/70k_checkcif.pdf]
